# Fumarase Deficiency and Its Effect on Infertility: A Case Series

**DOI:** 10.18502/jri.v24i3.13277

**Published:** 2023

**Authors:** Jessica Wesley Schwartz, Alexandra Peyser, Miriam Tarrash, Randi Heather Goldman

**Affiliations:** 1- Donald and Barbara Zucker School of Medicine at Hofstra/Northwell, Hofstra University, New York, USA; 2- Division of Reproductive Endocrinology, Department of Obstetrics and Gynecology, Northwell Health Fertility, North Shore University Hospital, New York, USA

**Keywords:** Fibroids, Fumarate hydratase, Genetic counseling, Hereditary leiomyomatosis and renal cell cancer (HLRCC), Infertility, Leiomyoma

## Abstract

**Background:**

Fumarase deficiency is an autosomal recessive condition characterized by severe neurologic abnormalities due to homozygous mutations in the fumarate hydratase (FH) gene. Heterozygous carriers of FH mutations have increased risk of developing uterine fibroids that can be associated with hereditary leiomyomatosis and renal cell cancer (HLRCC). The association between FH mutations and infertility remains uncertain. The objective of our study was to characterize the infertility diagnoses, treatments, and outcomes in women presenting to a fertility center who were found to be carriers of fumarase deficiency based on the presence of heterozygous FH mutations.

**Case Presentation:**

A retrospective case series was conducted including 10 women presenting to an academic fertility center who were found to be FH carriers based on genetic carrier screening. Of the 9 women who were engaged in further workup, 2 had imaging results consistent with uterine fibroids. One woman underwent hysteroscopic myomectomy prior to two courses of ovulation induction with timed intercourse (OI/TIC) followed by one successful cycle of IVF. Of the remaining patients, only 1 woman successfully delivered after a cycle of ovulation induction with intrauterine insemination (OI/IUI). Other patients pursuing OI/IUI, OI/TIC, or monitored natural cycles had unsuccessful experiences.

**Conclusion:**

Patients with infertility who are offered genetic testing should be screened for FH mutations, as the carriers are at risk of developing HLRCC-associated uterine fibroids, which can influence fertility and pregnancy. Additional research is needed to investigate the impacts of FH mutations on infertility.

## Introduction

FH mutation found through many genetic expanded carrier screening (ECS) tests has been shown to increase the likelihood of developing uterine fibroids in the heterozygous state (1, 2). Approximately 1 in 1000 people carry this mutation worldwide (3). FH mutations increase the risk of developing the autosomal dominant syndrome, hereditary leiomyomatosis and renal cell cancer (HLRCC) (2). Homozygous or compound heterozygous FH mutations result in fumarase deficiency, a rare autosomal recessive disorder characterized by severe neurologic and developmental deficits (1). Research has revealed that the fumarate hydratase enzyme coded by the FH gene is not only vital to the Krebs cycle but also acts as a tumor suppressor (2, 4). This proposed mechanism explains how FH mutations can increase the risk of developing leiomyomas of the uterus or skin, uterine leiomyosarcoma, and renal cell carcinoma (4, 5). Typically, FH carriers with HLRCC often present clinically with multiple early-onset leiomyomas of the skin and uterus, which are large in both number and size (4, 6).

Uterine fibroids are estimated to be present in 25% of women seeking medical assistance with reproduction and may be the sole cause of infertility in at least 2–3% of these women (7, 8). In addition to infertility, fibroids can cause pain, heavy or abnormal menstrual bleeding, and are associated with an increased risk of adverse obstetric outcome including recurrent pregnancy loss (9, 10). Risk factors associated with the development of fibroids include age, black race, family history, high body mass index (BMI), and genetic mutations (10). The location and size of fibroids, particularly the extent of endometrial cavity distortion, determine their impact on fertility (8). Submucosal fibroids negatively impact pregnancy as well as live birth rates and increase the risk of infertility and spontaneous abortion. Women with submucosal fibroids undergoing IVF have lower pregnancy, implantation, and delivery rates compared with women who do not have fibroids (10). Similarly, intramural fibroids may negatively impact fertility if they are large in size or cause distortion of the uterine cavity. In contrast, subserosal fibroids appear to have little to no impact on fertility or live birth rates (8, 10, 11).

To date, clinical data is lacking regarding fertility outcomes for carriers of fumarase deficiency, either due to the development of uterine fibroids or other uncharacterized metabolic effects. One prior case-control study examined pregnancy and fertility data and found no association in women with germline FH mutations or HLRCC (12). However, the cross-sectional nature of the study did not allow for a full investigation of the impact of FH mutations on infertility treatments and outcomes. The objective of this case series was to investigate the infertility diagnoses, treatments, and outcomes in women who are carriers of fumarase deficiency. Due to paucity of data, this study has provided initial insights on how heterozygous FH mutations are associated with infertility and therefore, how women seeking infertility treatment should be counselled.

## Methods

This was a retrospective case series including women observed at a single academic fertility center from January 2018 to July 2021. Women who presented for an infertility evaluation and underwent ECS as indicated for their infertility were the subjects of the study. Informed consent was obtained prior to screening and women were appropriately counselled by certified genetic counsellors both prior to and after screening. Women with FH mutations, detected upon genetic screening by Sema4 Elements™ ECS, were selected. The Sema4 Elements™ ECS tests for 283 genes via blood or saliva collection, with a self-reported analytical detection rate of 98% for FH mutations. All samples included in this study were processed by Sema4 laboratories. Patients who did not continue any further workup in our facility following genetic screening were excluded from the study.

Demographic and clinical variables including age, BMI, race, AMH levels, uterine length, fibroid size if present, infertility diagnosis, treatments, and outcomes of those treatments were obtained via chart review among patients identified with an FH mutation who continued further workup in our facility. Data was de-identified prior to reporting. Data reporting and analysis were primarily descriptive due to the observational nature of the study design.

This study was conducted in accordance with all guidelines set forth by the Institutional Review Board at the Feinstein Institutes for Medical Research at Northwell Health Fertility. Informed consent for genetic testing was obtained from all individuals undergoing testing, and the Institutional Review Board at the Feinstein Institutes for Medical Research at Northwell Health Fertility waived authorization for use of de-identified aggregate data (#21-0818). Individuals who opted out of this type of data use were excluded.

## Results

Of 5,841 patients genetically screened during the study period, 10 patients were found to be carriers of an FH mutation. One patient decided not to continue further workup or treatment with our practice and was therefore excluded from the analysis. Demographics and clinical variables for each patient, A–J, are shown in table 1. Fertility diagnoses, treatments, and outcomes for each patient are shown in table 2. There were 2 patients (A and G) whose fertility treatments were not followed by our facility. Of 9 patients reviewed, 8 had FH mutation, c.1431_1433dup, and 1 patient (B) had mutation c.521C>G. The median patient age was 33 years (range: 32–39 years) and most patients had normal ovarian reserve (median AMH: 2.74 *ng/ml*; range: 0.44–5.56 *ng/ml*). All patients in this cohort had a BM I above the normal range, with the median BMI in the over-weight category at 27.8 *kg/m*^2^ (range: 25.1–37 *kg/m*^2^). Primary infertility diagnoses were variable with the most common being unexplained (33%).

**Table 1.** Demographics and clinical variables

ID ^*^	**Age (y)**	**BMI (*kg/m*^2^)**	**Race**	**AMH (*ng/ml*)**	**Relevant family history**	**Uterine length (*cm*)**	**Uterine fibroids**
**A**	39	27.8	White	2.73	Mother: dysmenorrhea Sister: infertility, miscarriage	8	
**B ^+^**	38	34	White	0.44		8	1xIM, 3.9x4.8 *cm* 1xSS, 8x7.9 *cm*
**C**	33	32.7	Multiracial	5.42		8	Multiple IM, ≤5 *cm* 3xSM, removed, ≤3 *cm*
**D**	33	25.1	White	2.74	Mother: BRCA2+ Maternal grandmother: stage IV breast cancer, diagnosed at 49	5	
**E**	32	26	White	4.43		5.6	
**F**	33	27.5	White	5.56	Paternal aunt: infertility, miscarriage	4.9	
**G**	38	37	Black	1.02	Mother: breast cancer Maternal aunt: breast cancer	6.2	
**H**	32	25.1	White	4.31	Mother: breast cancer	7	
**J**	33	36.6	Unknown	1.61		6.2	

* All patients have FH mutation, c.1431_1433dup, except as otherwise noted.

+ Patient has FH mutation, c.521C>G

AMH: anti-Mullerian hormone, BMI: body mass index, IM: intramural, SM: submucosal, SS: subserosal

**Table 2.** Infertility diagnoses, treatments, and outcomes

ID ^*^	**Infertility diagnosis**	**Treatment**	**Outcomes**	**Antepartum complications**
**A**	RPL	IVF + PGT		
**B ^+^**	DOR + Male factor	IVF + PGT		
**C**	Unexplained	OI/TICx2 IVF + FET	CS at term due to breech presentation	IUGR
**D**	Elective egg freezing			
**E**	Unexplained	OI/IUIx2 OI/TICx1 OI/IUIx2		
**F**	PCOS	IUIx1	NSVD at term	GHTN IUGR
**G**	Tubal disease + Male factor	IVF		
**H**	Unexplained	NAT		
**J**	Same-sex couple	OI/IUI NAT OI/IUI		

^*^ All patients have FH mutation, c.1431_1433dup, except as otherwise noted.

+ Patient has FH mutation, c.521C>G

CS: Cesarean Section, DOR: Diminished Ovarian Reserve, FET: Frozen Embryo Transfer, IUI: Intrauterine Insemination, IVF: In Vitro Fertilization, NAT: Monitored Natural Cycle, NSVD: Normal Spontaneous Vaginal Delivery, OI: Ovulation Induction, PCOS: Polycystic Ovary Syndrome, PGT: Preimplantation Genetic Testing, RPL: Recurrent Pregnancy Loss, TIC: Timed Intercourse, GHTN: Gestational Hypertension

Of 9 FH carriers, 2 were found to have fibroids on ultrasound (B and C). Patient B was found to have two large fibroids, one intramural and one subserosal. She had planned a cycle of IVF and preimplantation genetic testing (PGT) for embryo banking. However, the cycle was canceled due to minimal follicular growth despite aggressive protocols. Patient C had several intramural fibroids, the largest being 5 *cm*, as well as three submucosal fibroids, the largest being 3 *cm*, which were resected by hysteroscopy prior to fertility treatment initiation. She initially attempted two cycles of ovulation induction with timed intercourse (OI/ TIC) which were unsuccessful before proceeding with IVF and frozen embryo transfer (FET). During egg retrieval, 24 oocytes were harvested, with 20 proceeding to intracytoplasmic sperm injection (ICSI). Finally, 15 embryos were successfully fertilized and 10 were frozen. The patient conceived from her first FET cycle and had a pregnancy complicated by intrauterine growth restriction (IUGR). Ultimately, she delivered via primary cesarean section at 37 weeks and 2 days secondary to breech presentation. The baby was born small for gestational age.

Of the 5 patients who did not have fibroids and whose treatments were followed in our facility, patient F with PCO-related infertility conceived after one cycle of ovulation induction with intrauterine insemination (OI/IUI). Her pregnancy was complicated by hypertensive disorder and IUGR. She had a normal spontaneous vaginal delivery (NSVD) at 37 weeks and 2 days, giving birth to an infant who was small for gestational age. Patients E, H, and J pursued only OI/IUI, OI/TIC, or monitored natural cycles with unsuccessful outcomes. Patient D was considering elective egg freezing but decided not to proceed with treatment (Table 2).

## Discussion

Of 5,841 patients screened in our practice during the study period, 10 patients were found to be carriers of fumarase deficiency (0.17%), which is greater than expected based on prevalence estimates of 1 in 1,000 (3). This difference may be due to the fact that our population presented with fertility concerns. Additionally, uterine fibroids were detected in 22% (2 out of 9) of women. While rates of fibroids in this cohort are similar to national estimates of women with fibroids seeking infertility care, this study was not powered to detect a difference. Due to the known link between FH mutations and fibroids associated with HLRCC, women seeking reproductive assistance undergoing comprehensive reproductive counselling and treatment including genetic carrier screening should be screened for FH mutations and counselled accordingly.

Genetic testing is the most accurate method of screening for risk of HLRCC, as it is almost always associated with a germline mutation (2). Though pathology reports of uterine fibroids show sporadic FH mutations in roughly 1% of patients, development of HLRCC with a sporadic mutation is rare (13). As of now, the only method of definitive diagnosis of HLRCC is genetic testing for germline mutations of FH, with a few proposed variations on major and minor diagnostic criteria (2). The major criteria currently accepted and outlined by Smit et al. and Schmidt and Linehan include multiple cutaneous leiomyomata with at least one histologic confirmation on biopsy. Minor criteria include solitary cutaneous leiomyoma with family history of HLRCC, pathologically confirmed early onset of type 2 papillary renal tumors, and multiple early-onset symptomatic uterine fibroids before the age of 40 (14, 15). Recent literature has shown that uterine leiomyomas with FH mutations do not always show consistent changes to cellular morphology nor are IHC stains particularly sensitive or specific in diagnosing HLRCC (13, 16). Therefore, pathologic examination may be best used with other clinical criteria when diagnosing HLRCC (13). Interestingly, presence of cutaneous leiomyomas is considered a common, specific, and early sign of HLRCC. However, one review demonstrated cutaneous leiomyomas present in only 71.5% of HLRCC cases, as compared to uterine fibroids in 83% of cases (17). Moreover, certain FH mutations have been associated with the development of uterine fibroids in the absence of cutaneous leiomyomas (18). Thus, the absence of cutaneous leiomyomas should not preclude consideration of HLRCC diagnosis when FH mutations are found on genetic carrier screening.

Thus, women who are found to be at risk for HLRCC via diagnostic criteria should be counselled about the risk of developing uterine fibroids. Stewart et al. conducted a case-control study of 105 women and found a 7.6-fold increased risk of developing uterine fibroids for those with an FH mutation as compared to those without. Moreover, these women are typically diagnosed with uterine fibroids at a younger age and with more associated symptoms, which may impact fertility (12). Submucosal and large intramural fibroids are especially known to negatively impact fertility and pregnancy outcomes (10). As surgical removal of submucosal fibroids is known to improve pregnancy and IVF outcomes, myomectomy may similarly improve fertility outcomes of women who are FH carriers (11). Women with existing fibroids should also be counselled about the likelihood of continued fibroid development that may interfere with fertility care.

Importantly, patients with a germline FH mutation should be advised about the risk of cancer. Uterine leiomyosarcoma has been shown to primarily affect FH carriers with Finnish heritage, and the overall lifetime risk of developing renal cell carcinoma for women with FH mutations has been estimated to be between 1.7% and 5.8% (3, 5). Development of renal carcinoma has been shown to appear later than uterine or cutaneous manifestations, which offers ample time for screening and intervention (6). A case report by Rivera-Cruz et al. describes a woman seeking fertility care who was diagnosed with HLRCC syndrome via germline testing and myomectomy tissue histopathology and immunostaining. This diagnosis enabled early detection of renal cell carcinoma in a family member. Furthermore, the patient elected to undergo IVF with PGT for monogenic disease and aneuploidy (PGT-MA) to minimize risk of passing on an FH mutation to her future children (19). Notably, the specific FH mutation may dictate individual risk of HLRCC diagnosis and phenotype. There has been a total of 363 FH mutations published, with only 225 thoughts to be pathogenic (18). The most commonly reported mutation, c.1431_1433dup, is currently not thought to be associated with an increased risk of developing renal cancer (20). Patients who are found to be at risk for HLRCC should be referred for screening, along with their family members. These patients should also be informed of options for IVF with PGT-MA as part of their fertility care, especially if a mutation is discovered during screening.

This descriptive study found that most patients who underwent OI/IUI were unsuccessful, and all attempts of OI/TIC and monitored natural cycles were ineffective. This may be due to the expected low success rates with these interventions and the low number of patients analyzed. The success rate for IUI in general population is about 10% for women aged 35–40 and less than 5% per cycle for women over 40 (21). In our study, OI/IUI was successful in only one patient, who had PCO-related infertility and no fibroids.

Despite the research available on FH carriers and HLRCC, there is limited data available on how FH mutations may affect fertility. Stewart et al. found that women with germline FH mutations or clinical HLRCC diagnoses experienced no negative effects on fertility or pregnancy (12). However, the authors pointed out that due to the cross-sectional nature of their study, more research was necessary. Our research was a longitudinal case series with highly detailed examination of fertility diagnoses, treatments, and outcomes in women with FH mutations. Limitations of our analysis include its descriptive nature and the small sample size due to the rarity of cases carrying an FH mutation. In cases of myomectomy, resected fibroids were not subject to further pathologic testing that would confirm association of fibroids with HLRCC. Additional research with rigorous study designs and precise statistical analysis should be conducted to ascertain whether FH mutations with or without HLRCC increase the likelihood of infertility and which treatments result in higher success rates.

## Conclusion

Women who are offered genetic screening for infertility treatment should be screened for FH mutations as they are at increased risk of developing uterine fibroids as a part of HLRCC. Submucosal or intramural fibroids, especially when recurrent, could interfere with fertility treatments and outcomes. Women should ideally undergo genetic testing, yet other diagnostic criteria including clinical findings, patient history, and histologic analysis can contribute to HLRCC diagnosis (14, 15). Patients with certain mutations should be counselled appropriately, particularly about IVF with PGT-MA as a fertility treatment option and about their possible referral for renal cancer screening. However, further research is needed to determine the likelihood of infertility in cases with fumarase deficiency and how the disorder affects treatment.
